# Decidual Natural Killer Cells: A Good Nanny at the Maternal-Fetal Interface During Early Pregnancy

**DOI:** 10.3389/fimmu.2021.663660

**Published:** 2021-05-12

**Authors:** Yuefang Liu, Shujun Gao, Yangjing Zhao, Hui Wang, Qiong Pan, Qixiang Shao

**Affiliations:** ^1^ Department of Clinical Genetics, the Huai'an Maternity and Child Clinical College of Xuzhou Medical University, Huai'an, China; ^2^ Reproductive Sciences Institute, Jiangsu University, Zhenjiang, China; ^3^ Department of Immunology, Jiangsu Key Laboratory of Medical Science and Laboratory Medicine, School of Medicine, Zhenjiang, China; ^4^ Jiangsu College of Nursing, School of Medical Science and Laboratory Medicine, Huai'an, China

**Keywords:** maternal-fetal interface, decidual natural killer cells, extravillous trophoblast cells, early pregnancy, functional dialogue

## Abstract

Decidual natural killer (dNK) cells are the tissue-resident and major subpopulation of NK cells at the maternal-fetal interface. It has been demonstrated that dNK cells play pivotal roles in pregnancy, including keeping maternal-fetal immune tolerance, promoting extravillous trophoblast (EVT) cell invasion, and driving uterine spiral artery remodeling. However, the molecular mechanisms haven’t been elucidated until recent years. In this review, we systemically introduce the generation, subsets, and surface or soluble molecules of dNK cells, which are critical for maintaining the functions of dNK cells. Further, new functions of dNK cells including well-controlled cytotoxicity, immunosurveillance and immunotrophism supporting *via* the cell-cell interaction between dNK cells and EVT cells are mainly focused. The molecular mechanisms involved in these functions are also illustrated. Moreover, pregnancy-associated diseases caused by the dNK cells abnormalities are discussed. It will be important for future investigations about the mechanism of maintenance of pregnancy and parturition and potential clinical applications of dNK cells.

## Introduction

The maternal-fetal interface, between the endometrium and extraembryonic tissue, plays pivotal roles in nutrient transportation, gas exchange, immune tolerance and protection for maintenance of pregnancy. The dynamic process of formation of the functional maternal-fetal interface mainly includes extravillous trophoblast (EVT) cell invasion, spiral arteriole remodeling, and establishment of the tolerant immune microenvironment (1–3). EVT cells migrate to the decidua tissue, invade the spiral artery wall and replace the spiral arteriole endothelial cells. Finally, the extensive physiological remodeling of the uterine spiral artery forms the vasculature with high-flow and low-resistance. The particular immune microenvironment at the maternal-fetal interface not only protects the fetus from the attack of maternal immune system, but also safeguards the mother from pathogens.

The maternal-fetal interface mainly constitutes trophoblast cells, decidual stromal cells (DSCs), different immune cells, such as decidual natural killer (dNK) cells, macrophages (Mφ), dendritic cells (DCs), T cells, B cells and NKT cells, and soluble factors derived from these cells (4). DNK cells are the most abundant immune cells population at the maternal-fetal interface. The ratio of dNK cells mostly reached 70% after 9-12 weeks of pregnancy in human beings. However, the percentages of macrophages and T lymphocytes are 10%-20% respectively (5). Traditionally, circulating NK cells not only play an important role in immunosurveillance but also maintain tolerance to self-tissues, which is called education (6). Self-major histocompatibility complex (MHC) class I molecules and specific MHC-I inhibitory receptors such as killer cell lectin-like receptor C1 (NKG2A) and killer cell lectin-like receptor subfamily A (Ly49) are responsible for NK education (7, 8). Interestingly, rather than immunosurveillance, dNK cells are specifically modified and mainly responsible for immune tolerance to the fetus, EVT cell invasion, and uterine spiral artery remodeling by interacting with EVT cells at the maternal-fetal interface (2). The molecular mechanisms have attracted much attention. However, they haven’t been fully elucidated for the dynamic complexity of multi-cell interactions. So far, the increasing evidence has truly lifted the veil of mystery of dNK cells. In this review, we focus on the frontier discoveries about the functions of dNK cells, including well-controlled cytotoxicity, immunosurveillance and immunotrophism from the interactions between dNK cells and EVT cells. The elucidated molecular mechanisms would help us understand pregnancy progress and provide new strategies for precision treatment of pregnancy-related diseases.

## Origin of Human dNK Cells

Unlike conventional circulating NK cells (CD16^+^CD56^dim^), most dNK cells are generally with CD56^bright^CD16^−^KIR^+^CD9^+^CD49a^+^ phenotype, lower cytotoxicity and higher cytokine secretion. The increasing evidence has revealed the origin of dNK cells and their homing mechanism. It has been demonstrated that decidual and peripheral CD16^-^ NK cells express high intensity of C-X-C chemokine receptor 4 (CXCR4) and are recruited to the decidua *via* CXC chemokine ligand 12 (CXCL12) (9, 10). Subsequently, CXCL12 was confirmed to be expressed only by EVT cells in decidua (10, 11). At decidua, the local microenvironment such as Galectin 9 (Gal-9)/T-cell immunoglobulin and mucin domain 3 (Tim-3) signaling induces the transformation of peripheral NK (pNK) cells into a dNK-like phenotype (12). However, another study showed that CD34^+^CD122^+^CD127^+^ hematopoietic precursors, present in decidua, were differentiated into dNK cells when co-cultured with DSCs *in vitro*. It has been demonstrated that IL-15 promotes the upregulation of Tsc1 and restricts exhaustive proliferation of immature NK cells, which is indispensable for the development and homeostasis of pNK cells (13–15). It has also been revealed that IL-15 is involved in the dNK cells differentiation process (16). DSCs express high levels of IL-15 and IL-15 receptor subunit α (IL-15Rα) complex, which pairs with its receptor consisting of IL-2Rγ and IL-2Rβ (expressed by dNK cells) (17). However, the molecular mechanism of the differentiation of CD34^+^ hematopoietic precursors into dNK cells driven by IL-15 has not been illustrated yet. The third possible differentiation route is that CD16^+^ pNK cells may migrate into uterine tissue and acquire the dNK phenotype under the influence of TGF-β and/or other factors (18). Taken together, it may have three origins of dNK cells: ① CD16^-^ NK cells are attracted by chemokines and immigrate from peripheral blood into decidua directly, where they differentiate into dNK cells under decidual microenvironment. ② dNK cells are differentiated from hematopoietic progenitor cells in the uterus. ③ dNK cells are directly converted from the CD16^+^ pNK cells.

## The Characteristics and Subsets of Human dNK Cells During Early Pregnancy

### Phenotypic and Functional Differences Between dNK and pNK Cells

The phenotype and function of dNK cells are different from pNK cells. Most dNK cells are generally with CD56^bright^CD16^−^KIR^+^CD9^+^CD49a^+^ phenotype, lower cytotoxicity and higher cytokine secretion (19–21). CD9 and CD49a are major markers of decidual tissue residency (22). PNK cells include the predominant subset of CD56^dim^ NK cells and the minor subset of CD56^bright^ NK cells (23). DNK cells resemble CD56^bright^ NK cells in terms of their CD56^bright^CD16^−^ phenotype and low cytotoxicity (24). On the other hand, similar with CD56^dim^ pNK cells, dNK cells are granulated and express killer immunoglobulin-like receptors (KIRs) (25). A comprehensive comparison of gene expression profiles in human dNK and pNK cells further illustrates the unique properties of dNK cells. Many surface receptor proteins such as killer cell lectin-like receptor C2 (NKG2C), killer cell lectin-like receptor C3 (NKG2E), Ly-49L, KIRs, Tim-3 and granzyme A are overexpressed in dNK cells (26, 27). Granulysin (GNLY), a novel cytolytic protein lytic against a variety of tumor cells and microbes, is also with a higher expression in dNK cells (28).

### dNK Cell Receptors

**Figure 1:**
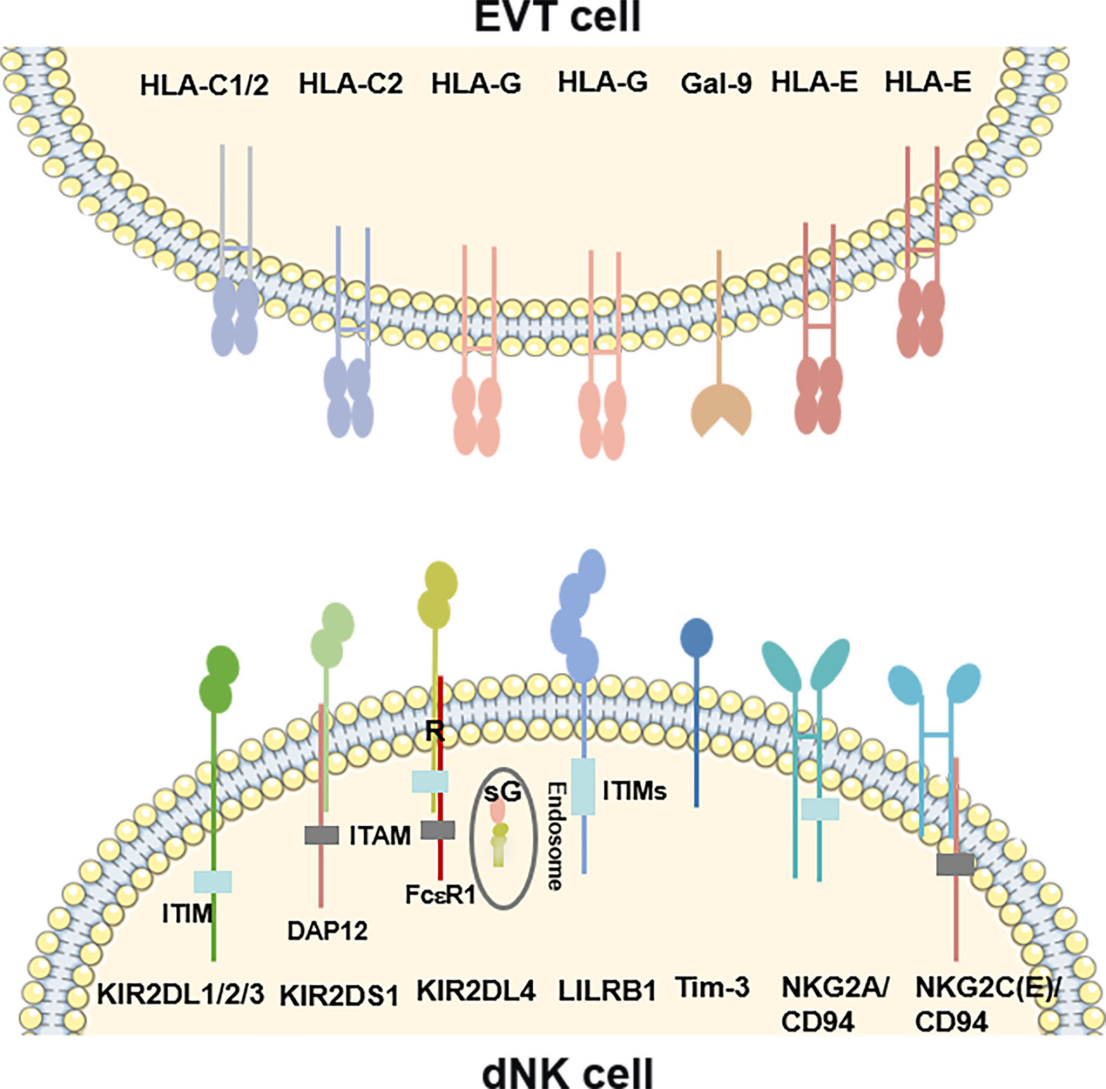
Overview of the ligand-receptor pairs between dNK cells and EVT cells. KIR2DL1, 2DL2 and 2DL3 are inhibitory receptors containing ITIMs, which bind with HLA-C on EVT cells. KIR2DS1 also interacts with HLA-C on EVT cells and activates downstream by combining with an ITAM–bearing adaptor DAP12. KIR2DL4, recognizing the HLA-G on EVT cells or sG from EVT cells, exhibits structural characteristics of both activating and inhibitory KIR by possessing a transmembrane R residue and a cytoplasmic ITIM. The transmembrane R binds with the adaptor protein FcϵR1 to transduce activating signals. KIR2DL4 is located in endosomes, and transferred to the surface of dNK cells when cultured with IL-2 *in vitro*. LILRB1 is also the HLA-G receptors containing four ITIMs in their cytoplasmic domains. NKG2 members form a heterodimer with CD94 and recognize HLA-E. NKG2A/CD94 is an inhibitory receptor owning ITIM in their cytoplasmic. While, NKG2C/E is activating receptors associated with the DAP12. Tim-3 interacts with its ligand Gal-9 on EVT cells. R, arginine; sG, soluble HLA-G; DAP12, DNAX-activating protein of 12; ITAM, Immunoreceptor tyrosine-based activation motif. ITIM, Immunoreceptor tyrosine-based inhibitory motif.

According to the functions, the NK cells receptors include two types receptors: activatory killer receptors (AKRs) and inhibitory killer receptors (IKRs). These receptors regulate the functions of dNK cells. However, based on the structure, the receptors can be divided into five families, such as killer cell immunoglobulin-like receptors (KIRs), leukocyte immunoglobulin-like receptors (LILRs), killer cell lectin-like receptors (KLRs), nature cytotoxicity receptors (NCRs) and T cell immunoglobulin and mucin domain (Tim). Binding with HLA-C and HLA-G, KIRs serve as critical regulators of dNK cells function (29–31) (**Figure 1**). The name of KIR is based upon the number of extracellular immunoglobulin-like domains (2D or 3D) and the length of the cytoplasmic domain (L for long and S for short). Killer cell immunoglobulin-like receptor, two Ig domains and long cytoplasmic tail 1 (KIR2DL1), 2DL2 and 2DL3 are IKRs, which bind with HLA-C to restrict cytotoxicity of dNK cells (**Figure 1**). These IKRs contain one or two immunoreceptor tyrosine-based inhibitory motifs (ITIMs), providing an inhibitory function (29). However, killer cell immunoglobulin-like receptor, two Ig domains and short cytoplasmic tail 1 (KIR2DS1) also interacts with HLA-C and activates downstream by combining with adaptor protein DAP12 (**Figure 1**) **(** 32). Immunoreceptor tyrosine-based activator (ITAM) within DAP12 triggers the Ras-RAF-MEK-ERK cascade and allows for activating function and cytokine production (29, 33). Different from 2DL1, L2 and L3, KIR2DL4 exhibits structural characteristics of both activating and inhibitory KIR by possessing both a charged transmembrane arginine residue and a single cytoplasmic ITIM (**Figure 1**) **(** 34). Instead of coupling with DAP12, the transmembrane arginine binds with the adaptor protein FcϵR1 and induces the Ras-RAF-MEK-ERK cascade by ITAM to transduce activating signals (33). In contrast, when the ITIM domain recruits the Src homology domain containing tyrosine phosphatase 2 (SHP-2), it has the potential to mediate negative signals (29). KIR2DL4 is located in endosomes, activated and transferred to the surface of dNK cells when cultured with IL-2 *in vitro* (**Figure 1**) **(** 35–37). Following the introduction above, we can summarize that there are two types of KIRs: KIR A has seven KIR genes, encoding six inhibitory receptors and an activating KIR2DS4. In contrast, the KIR B contains many additional KIRs, most of which encode activating receptors (38). LILRB1, a member of the LILRs family, is also the HLA-G receptors containing four ITIMs in their cytoplasmic domains (**Figure 1**) **(** 39). It has been revealed that dNK cells is one of source of LILRB1 in placenta (17, 20). KLRs family are composed of NKG2 members with CD94 and form a heterodimer and recognize HLA-E. NKG2A/CD94 is an IKR owning ITIM in their cytoplasmic (**Figure 1**). While, NKG2C and NKG2E are AKRs associated with an ITAM-bearing adaptor molecule DAP12 (**Figure 1**) **(** 17, 40, 41). NKG2D is an AKR and a unique member of the NKG2 family that does not bind to CD94. NKG2D forms the homodimer and recognizes the MHC class I chain-related molecules A/B (MIC A/B) (42). For the short of intracellular portion and lack of ITM, NKG2D transduces the activated signal *via* the intracellular homodimer of DAP-10 (mice) and/or DAP-12 (human and mice) containing ITAM, and initiates NK and T cell-mediated cytotoxicity (43). NCRs family consists of three type I transmembrane receptors, termed NKp46, NKp44 and NKp30, also known as NCR1, NCR2 and NCR3 (44). While, there is a unique NCR termed NKp80 expressed on human NK cells (45). There are all compose of C2-type Ig-like domain (NKp46 has two, but NKp30 and NKp44 have only one) and followed by a stalk region. NCRs are AKRs type receptor and lack the ITAM in the intracellular, transducing the signal via CD3ζ and/or FcγR (NKp46 and NKp30), or DAP12 (NKp44), respectively. However, NKp44 contains an ITIM sequence in cytoplasm and transduces inhibitory signaling in some situations (44). The ligands of NCRs are not MHC molecules such as viral hemagglutinins, B7-H6, human cytomegalovirus pp65, heparan sulphates and proliferating cell nuclear antigen (PCNA), etc. (46), but have not been fully revealed. NCRs bind to their ligands and regulate the functions such as cytotoxic effect and cytokine derived from NK cells. Tim-3, the main member of Tim family expressed by dNK cells, has been implicated both in activation and inhibition of immune responses upon stimulation by its ligand Gal-9 (**Figure 1**) **(** 47).

### The Subsets of dNK Cells

Recently, three main dNK cell subsets (dNK1, dNK2 and dNK3) were identified by single-cell RNA-sequencing (17). Heat map of gene expression in decidua significantly distinguished these three dNK cell subsets. The dNK1 cells express higher levels of IKRs (KIR2DL1, KIR2DL2 and KIR2DL3) and AKRs (KIR2DS1 and KIR2DS4) (17). LILRB1 is expressed only by the dNK1 subset (48). Both dNK1 and dNK2, but not dNK3, express NKG2C, NKG2E as well as NKG2A (**Table 1**) **(** 17). DNK1 contains more cytoplasmic granules (perforin, GNLY, granzyme A and granzyme B) than dNK2 and dNK3 (17). Tim-3 is expressed with a high level on dNK1 but low on dNK2 (**Table 1**) **(** 17). dNK3 expresses a high level of KLRB1 and without KIRs (**Table 1**) **(** 17). Other defining surface markers on the three dNK cell subsets are also shown in **Table 1**.

**Table 1:** The surface markers and KIR receptors of the three dNK cells subsets.

		dNK1	dNK2	dNK3
Surface Markers		CD39, CYP26A1, B4GALNT1, TIM3^high^	ANXA1, ITGB2 TIM3^low^	ITGB2, CD160, KLRB1, TIGIT, CD103
Receptors	Binding with HLA-C	KIR2DL1, IR2DL2, KIR2DL3, KIR2DS1, KIR2DS4		–
	Binding with HLA-G	LILRB1		–
	Binding with HLA-E	NKG2A (KLRC1)	NKG2A	–
		NKG2C (KLRC2)	NKG2C	
		NKG2E (KLRC3)	NKG2E	

B4GALNT1, β-1,4-N-acetyl-galactosaminyltransferase 1; CYP26A1, cytochrome P450 family 26 subfamily a member 1; ANXA1, annexin a1; TIM3, T-cell immunoglobulin and mucin domain 3; ITGB2, integrin subunit β2; KLRB1, the killer cell lectin-like receptor B1; TIGIT, T cell immunoreceptor with Ig and ITIM domains; HLA-C/G/E, major histocompatibility complex, class I, C/G/E; KIR2DL1/L2/L3, killer cell immunoglobulin like receptor, two Ig domains and long cytoplasmic tail 1/2/3; LILRB1, leukocyte immunoglobulin like receptor B1; KIR2DS1/S4, killer cell immunoglobulin like receptor, two Ig domains and short cytoplasmic tail 1/4; NKG2A/2C/2E, killer cell lectin-like receptor C1/C2/C3.

In addition to the surface molecules, there are many soluble molecules secreted by dNK cell subsets, such as chemokines, granulocyte-macrophage colony-stimulating factor (GM-CSF), interferon γ (IFN-γ), vascular endothelial-derived growth factor (VEGF), angiopoietin (Ang), placental growth factor (PLGF), soluble Fas ligand (sFasL), matrix metalloproteinase (MMP-2), MMP-9 and transforming growth factor-beta (TGF-β) (17, 49, 50). The surface or soluble molecules, derived from dNK cell subsets, bind to specific receptors or ligands on EVT cells and perform their functions. The functional differences between dNK cell subsets are listed in **Table 2**.

**Table 2:** The surface or soluble molecules from dNK cells subsets and their receptors/ligands on EVT.

Function	dNK1-EVT	dNK2-EVT	dNK3-EVT
Immunomodulation	KIRs-HLA-C/G NKG2A/NKG2C/NKG2E -HLA-E	NKG2A/NKG2C/NKG2E -HLA-E	KLRB1-CLEC2D
	TIIM3-Gal-9	TIIM3-Gal-9	TIGIT-PVR
	LILRB1-HLA-G	–	
	CD39-CD73		
EVT cells invasion	CSF1-CSF1R		CXCR4-CXCL12
	XCL1 (low)/XCR1	XCL1 (medium)/XCR1	XCL1 (high)-XCR1
			CCL5-CCR1

Gal-9, Galectin 9; CLEC2D, C-type lectin domain family 2, member D; PVR, poliovirus receptor; CSF1, Colony-stimulating factor 1; CSF1R, Colony-stimulating factor 1 receptor; XCL1, Lymphotactin; XCR1, Lymphotactin receptor; CXCR4, C-X-C chemokine receptor 4; CXCL12, CXC chemokine ligand 12; CCL5, C-C chemokine ligand 5; CCR1, CC Chemokine receptor 1.

### EVT Cells

The cytotrophoblasts (CTBs) differentiate into either invasive EVT cells or syncytiotrophoblast (STB) depending on the signals they receive (51). EVT cells are the invasive cells that migrate deeply into decidua and contact with maternal dNK. Unlike most cells, EVT cells are devoid of HLA-A and HLA-B expression, while expressing HLA-C, HLA-G and a low level of HLA-E (52)(**Figure 1**). HLA-C is classified into HLA-C1 and HLA-C2 based on natural amino acid at position 80 of the HLA-C heavy chain, where HLA-C1 has an asparagine and HLA-C2 has a lysine (53). HLA-C1 is recognized by KIR2DL2 and KIR2DL3, while HLA-C2 is a ligand for KIR2DL1 and KIR2DS1 (**Figure 1**). In comparison with HLA-C1, HLA-C2 combines more closely with homologous KIR (54). Membrane-bound HLA-G and soluble HLA-G are both synthesized by EVT cells. HLA-G, firstly discovered in 1982 by Orr, is only expressed by EVT cells (55, 56). It has a lower level of polymorphism since there are only 51 alleles encoding 16 different proteins (57). With trophoblast-specific expression and a lower degree of polymorphism, HLA-G might play an important role at the maternal-fetal interface. Soluble HLA-G derived from early embryos is pivotal for successful implantation and might be a predictor of increased pregnancy rate following *in-vitro*-fertilization (IVF) (58). After embryo implantation, both membrane-bound HLA-G and soluble HLA-G induced immune tolerance, promotes spiral artery remodeling and fetal growth (59). Other surface and soluble molecules, such as Gal-9, chemokines and pro-angiogenic factors, are also expressed by EVT cells. The interactions of some molecules with dNK cells subsets are listed in **Table 2**. Pro-angiogenic factors secreted by EVT cells involved in the epithelial-mesenchymal transition (EMT), invasion and spiral arterioles remodeling are also identified (**Table 3**) **(** 32).

**Table 3:** Cytokines involved in the EMT, invasion and spiral arterioles remodeling.

Cytokines	Function
TGF-β1	Suppress immune responses, EMT, spiral arterioles remodeling
PAPPA	Encode metalloproteinases
MMP2/3/10/11/12/14/15/25/28	EMT, spiral arterioles remodeling
FasL	Spiral arterioles remodeling
TRAIL	Spiral arterioles remodeling
VEGF	Spiral arterioles remodeling
Ang 1/2	Spiral arterioles remodeling

TGF-β1, transforming growth factor beta 1; PAPPA, pregnancy-associated plasma protein A; MMP, matrix metalloproteinase; FasL, Fas ligand; TRAIL, tumor necrosis factor-related apoptosis-inducing ligand; VEGF, vascular endothelial growth factor; Ang 1/2, angiotensin 1/2.

## The Functional Dialogue Between dNK Cells and EVT Cells at Maternal-Fetal Interface

### HLA-G Cycle and Gal-9/Tim-3 Signal Control the Cytotoxicity of dNK Cells

Although there are abundant cytolytic granules (similar RNA levels of granzyme B and perforin in dNK and CD56^dim^ pbNK, and higher level of granzyme A and GLNY in dNK) (26), dNK cells have a low cytotoxic effect on K562 cells, which are the typical sensitive target cells (MHC Class I negative cells) of pbNK (24). Early literature had reported that interaction between CD94/NKG2A receptor and its HLA-E prevented dNK cell cytotoxicity in a healthy pregnancy (60). In recent years, other mechanisms controlling cytotoxicity of dNK cells have been discovered.

**Figure 2:**
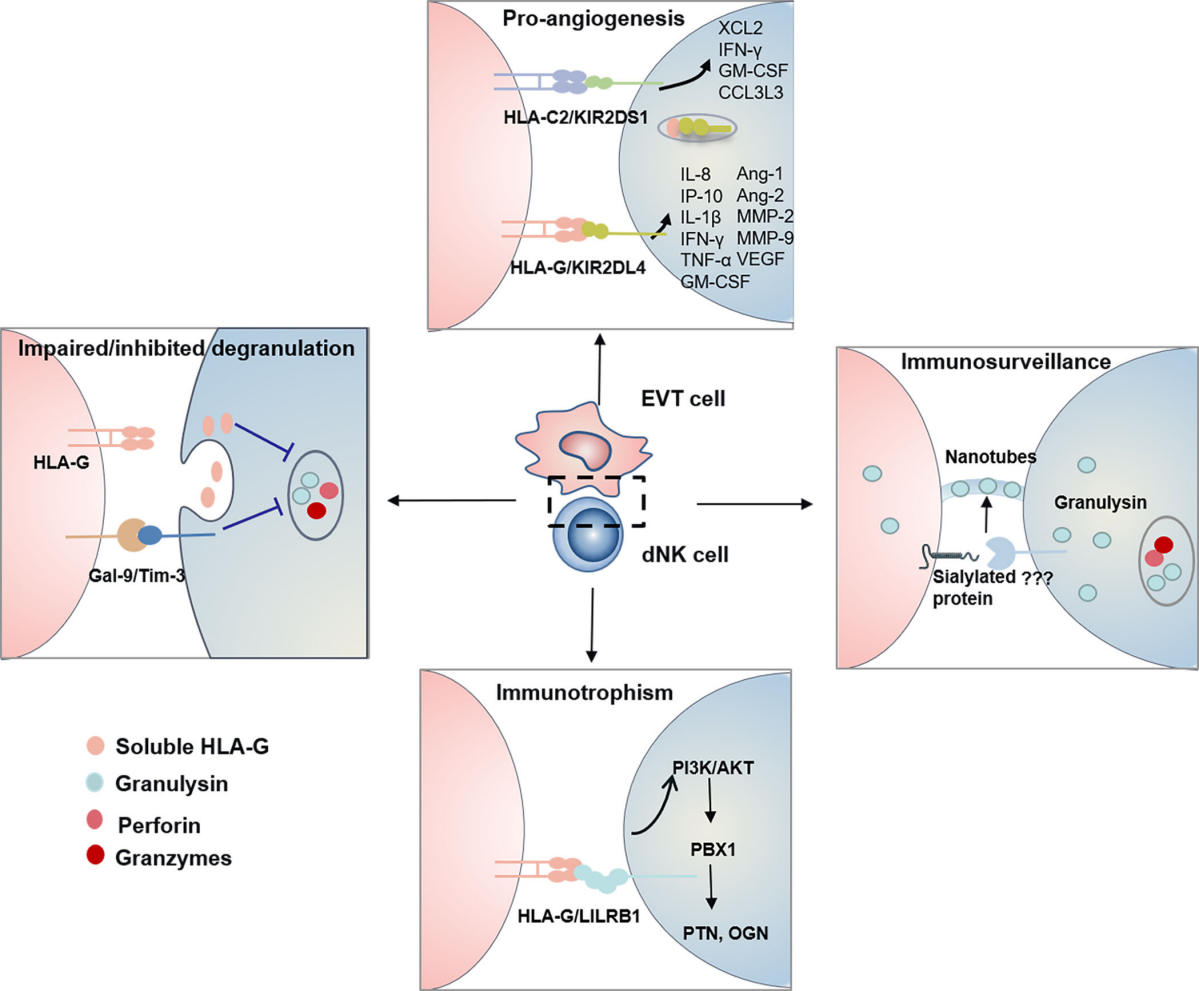
The functional dialogue between dNK cells and EVT cells at the maternal-fetal interface. HLA-G Cycle and Gal-9/Tim-3 signal inhibit/impair degranulation process and control the cytotoxicity of dNK Cells. Human dNK cells selectively transfer GNLY but not granzymes *via* nanotubes to EVT cells. Transferred GNLY kills intracellular bacteria without killing the EVT cells. Sialylated protein on the target cell promotes nanotube formation, while its receptor on dNK cells need further research. KIR/HLA allorecognition system (KIR2DS1-HLA-C2 and KIR2DL4-HLA-G) drives EVT cells invasion and uterine artery remodeling *via* synthesizing a series of soluble factors. HLA-G-LILRB1 signal activates the PI3K-AKT pathway and drives the expression of transcription factor PBX1. Furthermore, PBX1 upregulates the transcription of the GPF including PTN and OGN and promotes fetal development.

Tilburgs et al. found an interesting HLA-G cycle phenomenon: upon interaction with EVT cells, dNK cells display trogocytosis, endocytosis, degradation, and finally reacquisition of HLA-G, endowing a transient acquisition of cytolytic capacity (61) (**Figure 2**). The dNK cells are cytotoxic in the absence of HLA-G, while non-cytotoxic in the presence of HLA-G (61). IL-15, IL-2 and IL-12 could interrupt the HLA-G cycle and increase the cytotoxicity of dNK cells (32, 61). KIR2DS1 may contribute to the uptake of HLA-G by dNK cells (61). Thus, the HLA-G cycle may provide a direct inhibition of cytotoxicity (**Figure 2**).

Tim-3 is expressed on more than 60% of human dNK cells and significantly higher than pNK cells (12). Gal-9 is present in DSC (17) and approximately 75% of trophoblast cells (12). TGF-β1 promotes the conversion of pNK cells to dNK cells and upregulates Tim-3 expression (18, 27). Contradictory with recent finding that Tim-3^+^ dNK1 cells expressed higher level perforin (17), previous research reported that Tim-3^+^ dNK cells were with immune tolerance characterized by expressing higher Th2-type cytokine IL-4 and lower level perforin (12). It is known that Gal-9/Tim-3 signal modulates the Th1/Th2 balance (62), while abnormal expression of Tim-3 in NK cells is related to many diseases (63). Recently, it has been evidenced that Gal-9/Tim-3 signal pathway inhibits the cytotoxicity of human dNK cells *via* impairing NK degranulation process (**Figure 2**) (17, 27). A decreased percentage of Tim-3^+^ dNK cells with increased Th1-type cytokines was observed in human and mice miscarriages (12). While, the excessive activation of Tim-3/Gal-9 is related to recurrent spontaneous abortion and preeclampsia (64, 65). Therefore, the significance of Gal-9/Tim-3 signal for dNK cells has remained mostly unknown.

### Granulysin in dNK Cells Provide Immunity to Infected EVT Cells

Infections have been reported to be a major cause of spontaneous abortions. Many investigations on controlling the cytotoxicity of dNK cells have been performed. However, it has been ignored how dNK cells protect the placenta from viral and bacterial infections. Traditionally, perforin, granzymes and GNLY are the cytotoxic effectors in NK cells, which cooperatively kill the infected cells (66). DNK cells have been reported to clear human cytomegalovirus (HCMV)-infected DSCs *via* perforin, granzymes and GNLY (61, 67). The mechanism shows that IL-15 inhibits the HLA-G cycle and promotes degranulation, and serves to ensure the NK control of virus infection at the interface (61). It has been revealed that the percentage of KIR2DS1^+^ dNK cells positively correlated with degranulation level. They produce more IFN-γ, TNF-α, and GM-CSF than KIR2DS1^−^ dNK on the response to HCMV-infected DSC (67). However, HCMV-infected EVT cells, cannot be cleared in this way, would result in virus-induced placental pathology and development of complications later in pregnancy. Recently great progress has been made in how dNK cells protect placental from infection while maintaining fetal tolerance. It has been elucidated that dNK cells are dependent on direct contact to kill *Listeria monocytogenes* (Lm) within EVTs and not on perforin, granzymes, or degranulation. Human dNK cells selectively transfer GNLY but not granzymes *via* nanotubes to EVT cells. Transferred GNLY kills intracellular Lm without killing the EVT cells. Sialylated protein on the target cell promotes nanotube formation, while its receptor on dNK cells need further research (68) (**Figure 2**).

### KIR/HLA Allorecognition System Drives EVT Cells Invasion and Uterine Artery Remodeling

The spiral artery remodeling (SAR), essential for promoting blood flow to the placenta and fetal development, is mainly accomplished by dNK cells and EVT cells. DNK cells accumulate around the spiral artery (SA) and initiate the SAR before EVT invasion. Subsequently, EVT cells invade the SA, induce the apoptosis of vascular smooth muscle cells (VSMC) and endothelial cells, and replace the endothelium. These processes have been summarized into “trophoblast-independent” and “trophoblast-dependent” stages (69–71). During the trophoblast-independent stage of uterine arterial remodeling, dNK cells surround SA and synthesize a series of soluble factors to induce EVT invasion and initiate destabilization of vascular structures including IL-8, IFN-γ-induced protein 10 (IP-10, is also known as CXCL10), GM-CSF, PLGF, IFN-γ, TNF-a, VEGF, Ang-1/2 and MMP-2/9.

IL-8 and CXCL10 are both members of the CXC subfamily of chemokines. After purifying human dNK cells and EVT cells, the microarray analyses were performed and found that IL-8 and CXCL10 were released by dNK cells, while C-X-C chemokine receptor 1 (CXCR1) and CXCR3 were produced by EVT cells (72). Subsequently, it was demonstrated that the CXCR1-IL-8 and CXCR3-CXCL10 pathways were responsible for the invasion of EVT cells (72). GM-CSF has been proved to regulate pre-implantation embryo, placental trophoblast cells invading and the maternal immune tolerance in mice and human pregnancy (73). PLGF promotes invasion of primary CTBs, while TNF-α and IFN-γ inhibit trophoblast migration and invasion, which cooperatively controls the depth of EVT invasion (49). IFN-γ, Ang-1/2, VEGF and MMP-2/9 are capable of stimulating VSMC destabilization (74, 75). KIR2DS1-HLA-C2 and KIR2DL4-HLA-G are the two main allorecognition systems responsible for the production of these soluble factors (**Figure 2**).

### KIR2DS1-HLA-C2

Interaction between KIR2DL1 with HLA-C2 is associated with the risk of pregnancy disorder, whereas KIR2DS1 protects from pregnancy disorders *via* interacting with HLA-C2. Robust evidence, although not consistent, has demonstrated that loss of activating KIR combined with fetal HLA-C2 increases the risk of preeclampsia (38, 76). The presence of activating KIR correlates with an increased birth weight (77). However, the molecular mechanism explaining this protective function is not fully revealed. To clarify the roles of KIR2DS1-HLA-C2 interaction in the cytokines releasing, KIR2DS1 single positive dNK cells were isolated and co-cultured with HLA-C2^+^ cells, and the microarrays assay was performed. It was found that KIR2DS1-HLA-C2 significantly promoted the secretion of IFN-γ, GM-CSF, chemokines XCL2 and CCL3L3 from dNK cells (78) (**Figure 1**). These findings provide a molecular mechanism explaining how the KIR2DS1-HLA-C2 are beneficial for placentation. However, in another study, KIR2DS1^+^ dNK cells stimulated by HLA-C were not found to generate GM-CSF (79). Therefore, the molecular mechanism of KIR2DS1 protection remains to be further explored.

### KIR2DL4-HLA-G

KIR2DL4 is located in endosomes and expressed on the surface of NK cells when stimulated with IL-2. Earlier studies have indicated that KIR2DL4 induced unique cytokine/chemokine, including pro-inflammatory/pro-angiogenic cytokines, such as IFN-γ, TNF-α, IL-1β, IL-6, IL-8, CXCL10, PLGF and VEGF (80). However, little was known about the signal transduction pathways triggered by surface and endosome KIR2DL4. It has been clarified that for surface KIR2DL4, cytokine and chemokine responses are regulated by different pathways (80). Weak cytolytic activity, calcium responses and secretion of IFN-γ or IL-8 mainly rely on transmembrane arginine residue by binding with transmembrane FcϵR1, which are largely abrogated by mutation of the transmembrane arginine. In addition, KIR2DL4 can activate MAPKs and NF-κB signaling pathways and facilitate the expression of MIP1α (also known as CCL3) through an unknown domain, which is not affected by transmembrane arginine region. HLA-G combined with the surface KIR2DL4 on dNK cells induces upregulation of CXCL10, PLGF and VEGF (81). While in the endosome, HLA-G, up-taken by dNK cells, combines with KIR2DL4, which initiates the production of IFN-γ, TNF-α, IL-1β, IL-6 and IL-8 *via* PKCs-Akt-NF-κB signaling pathway (**Figure 2**) **(** 35). In addition to cytokines mentioned above, related studies found that dNK cells also produced Ang-1/2, VEGF, FasL and MMP2/9 to promote vascular remodeling (49).

### LILRB1-HLA-G Signal Takes Part in Immunotrophism

LILRB1 has a higher affinity to dimeric HLA-G than that of monomeric forms (82). Recent research found that HLA-G-LILRB1 signal served as a stimulus for growth-promoting factors (GPF)-secreting function of dNK cells, which was critical for the development of blood vessels, bone, cartilage, neurite, and cardiac tissue in the fetus (20). The lack of GPF-secreting NK cells impairs fetal development and results in fetal growth restriction in humans and mice (20). The transcription factor nuclear factor interleukin-3-regulated (Nfil3) controls the number of GPF-secreting NK cells (20). It has revealed that intracellular HLA-G-LILRB1 signal activates the PI3K-AKT pathway and drives the expression of transcription factor pre-B-cell leukemia homeobox 1 (PBX1). Furthermore, PBX1 upregulates the transcription of the GPF including pleiotrophin (PTN) and osteoglycin (OGN) and promotes fetal development (83) (**Figure 2**). Interestingly, a subpopulation of dNK cells with highly NKG2C and LILRB1-expressing was identified in repeated pregnancies and termed as pregnancy-trained decidual natural killer cells (PTdNK cells) (84). Compared with dNK from women with first pregnancy, HLAG-LILRB1 in PTdNK cells promotes greater levels of IFN-γ and VEGFα, which is essential for angiogenesis and appropriate placental bed formation. The epigenome of PTdNKs were found to have a unique chromatin modification, allowing for active transcription (84). These results suggested the role of HLA-G-LILRB1 in promoting placentation in repeated pregnancies.

## dNK Cells and Pathological Pregnancy

### Preeclampsia

During normal pregnancy, the spiral arteries in the endometrium and decidua are reconstructed into low-pressure blood vessels by EVT cells and dNK cells. During the second trimester, pregnancy complications occur when uterine spiral artery remodeling is limited, such as preeclampsia (85, 86). Preeclampsia is a systemic disease involving multiple organs with high blood pressure and proteinuria and accurses following the 20-week period of pregnancy and threaten the life of both the mother and the fetus (87). Many factors have been reported to be correlated with preeclampsia, including environment, genes and immune (88–90). The increasing evidence demonstrated that soluble endoglin (sENG) and soluble fms-like tyrosine kinase-1 (sFlt-1) are anti-angiogenic factors that act against VEGF and TGF-β and contribute to preeclampsia in the placenta (91, 92). However, molecular mechanisms leading to the upregulation of sENG and sFlt-1 have not been fully understood.

The mismatch between dNK cells and EVT cells at the maternal-fetal interface is another risk closely related to the development of preeclampsia. The combination of two maternal KIR A haplotypes (KIR AA) and fetal HLA-C2 has been demonstrated more common in preeclampsia by comparing 200 cases of preeclampsia patients with 201 cases with a normal pregnancy in British women (38). The preeclampsia patients from China also show a higher frequency of KIR AA (93, 94). For Mexico preeclampsia patients, KIR AA is even predominated (95). Activating KIR is capable of stimulating dNK cells and promoting the angiogenesis and immune response, contributing to a healthy pregnancy. Whereas inhibiting KIR is likely to lower the secretion of cytokines, thereby causing preeclampsia. However, some contradictory findings were observed as well. In one study including 324 couples consisting of Japanese women (with the highest frequency of KIR AA alleles and lowest frequency of HLA-C2 alleles) and caucasian men(with a moderate frequency of KIR AA and HLA-C2 alleles), the incidence of preeclampsia was similar to that in couples consisting of Japanese women and Japanese men (96). In another study, 518 Danish pregnant women were grouped into severe preeclampsia and normal control, and no correlation existed between maternal KIR AA and fetal HLA-C2 (97).

### Recurrent Spontaneous Abortion

The American Society for Reproductive Medicine (ASRM) defines recurrent spontaneous abortion (RSA) as two or more pregnancy losses (98). RSA is one of the major challenges in reproductive medicine and affect 1%-2% of reproductive women (99). Chromosomal anomalies account for 50% of RSA (100). The remaining factors include immunity, environment, thrombosis and uterus. A consistent conclusion on the relationship between the number of NK cell and RSA has not been achieved currently. Compared to the normal pregnancy, women with RSA are characterized by a decreased amount of inhibitory NKG2A^+^ dNK cells and an increase in the number of decidual CD56^+^ cells with CD161, granzyme B and IFN-γ (101). In contrast, another study revealed that dNK cells showed no difference in women with RSA compared with controls (102). The abnormal function of dNK cells at the maternal-fetal interface has been confirmed to be involved in RSA. Guo et al. revealed that secretion function of human dNK cells, involved with VEGF, IL-8 and CXCL10, was impaired in RSA, which led to the inhibited pro-invasion and pro-angiogenesis functions of dNK cells. A high level of miR-133a in RSA villi downregulated HLA-G expression in EVT cells, which downregulated KIR2DL4-HLA-G signaling and suppressed the secretion function of human dNK cells (81). Molecular mechanism regulating the expression of HLA-G on dNK cells need further research.

## Conclusions

Currently, reproductive health problems have gradually become a hot issue. A variety of major diseases, including preeclampsia and RSA, cause family members to suffer from childlessness. At present, the understanding of the mechanisms of these pregnancy diseases is still quite limited, leading to a lack of scientific and effective early prediction and intervention strategies for the diseases.

In this review, we introduced new functions of dNK cells including well-controlled cytotoxicity, immunosurveillance, and immunotrophism from the interactions between dNK cells and EVT cells. Molecular mechanisms underlying multifunctional dNK cells involve HLA-G cycle, Gal-9/Tim-3, KIR-HLA and LILRB1-HLA-G signals. The interactions among these molecules finally promote successful pregnancy *via* inhibiting degranulation, transferring GLNY and inducing cytokines, such as IFN-γ, GM-CSF, VEGF, IL-8, CXCL10, PLGF, PGF and PTN. In summary, this review proposes suggestions on the possibility of dNK cells and related molecules as the targets of investigation and therapy for pregnancy complications.

## AUTHOR CONTRIBUTIONS

YL, SG, YZ, and HW contributed to the conception. YL collected relevant studies and wrote the manuscript. HW, QP and QS were the funding recipients and contributed to the revision, and final approval of the manuscript. All authors contributed to the article and approved the submitted version.

## FUNDING

The present study was supported by grants from Chinese National Natural Science Foundation Grant (grant nos.82071738, 81671541, 81701545, and 31400773), Clinical Medicine Science & Technology Project of Jiangsu Province of China (grant no. BL2013024) and Open project of Jiangsu Province Key Laboratory (XZSYSKF202002).

## Conflict of Interest

The authors declare that the research was conducted in the absence of any commercial or financial relationships that could be construed as a potential conflict of interest.
